# Universal evidence and contextual pharmacotherapy

**DOI:** 10.1093/ehjcvp/pvag065

**Published:** 2026-09-22

**Authors:** Bianca Rocca

**Affiliations:** Department of Medicine and Surgery, LUM University, 70010 Casamassima (Bari), Italy

**Figure:**
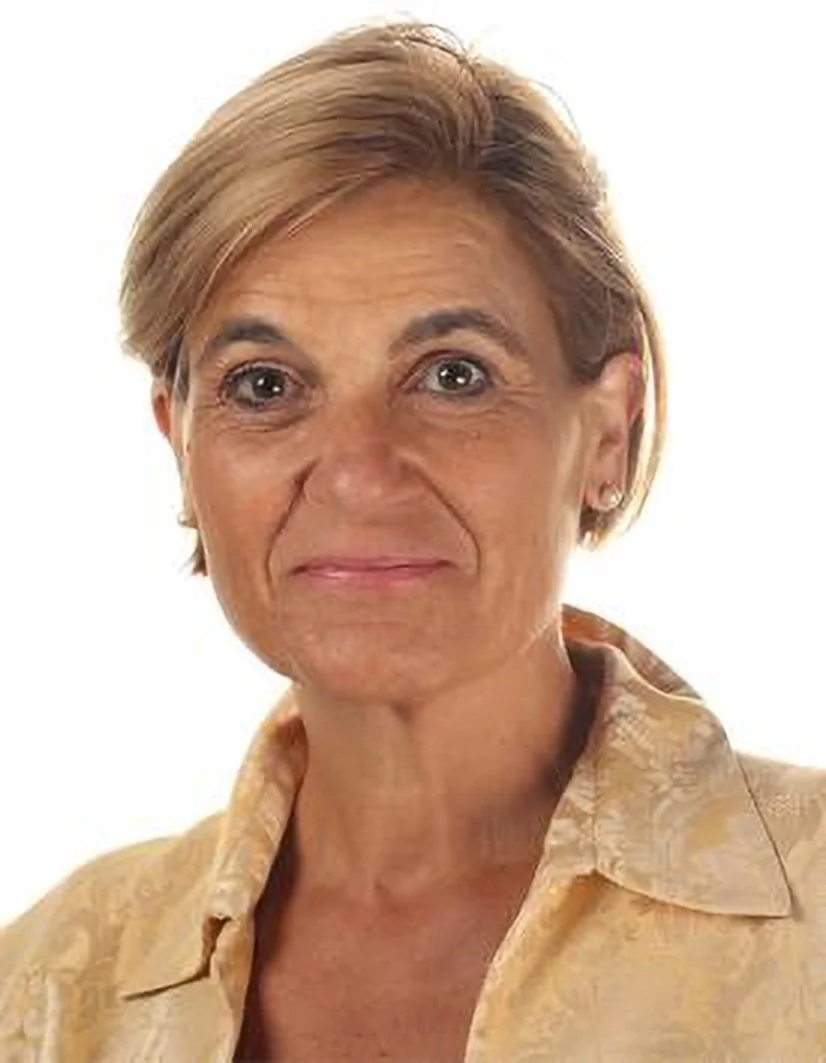


Cardiovascular pharmacotherapy is traditionally guided by a simple paradigm: evidence generated from randomized clinical trials is translated into guidelines, implemented in clinical practice, and expected to yield predictable benefits. The contributions assembled in this issue suggest a more complex reality. Across diverse clinical settings, they indicate that therapeutic success depends not only on the intrinsic pharmacological properties of an intervention but also on the context in which evidence is generated, interpreted, prescribed, implemented, and experienced by individual patients.

Omerovic *et al.*^1^ challenge the assumption that randomized trials necessarily generate universally transferable truths. The authors discuss two divergent trials of ß-blocker agents after myocardial infarction (MI), the Treatment with Beta-Blockers after Myocardial Infarction without Reduced Ejection Fraction (REBOOT-CNIC) with 8505 participants from Spain and Italy, which found no significant reduction in death or major cardiovascular events as well as the combined Norwegian BETAMI and the Danish DANBLOCK trials with 5574 participants from Norway and Denmark, which reported a statistically significant, 15% relative reduction in its larger composite endpoint including MI, death, unplanned revascularization, ischaemic stroke, heart failure hospitalization, and malignant ventricular arrhythmia. The authors rigorously analyse the entire deterministic causal chain, resist the tempting reconciliation of inclusive meta-analyses, overcome a reductionist approach and introduced the concept of ‘causal environment’. In this framework, treatment effects may depend on pharmacokinetic (PK) drug-specific characteristics even within the same class, healthcare systems, geography, including climate and circadian stressors, and clinical settings. Thus, evidence cannot be entirely separated from the environment in which it is generated and applied. Registry-based trials and phase IV studies may represent important tools for assessing precision causality within the ‘causal environment’.

Marcucci *et al.*^2^ further discuss the same issue of identifying the ‘Truth’ from an interesting, complementary perspective. Drawing on the Grading Recommendations Assessment, Development and Evaluation (GRADE) framework, they argue that apparent inconsistencies between studies should not be interpreted only through conventional measures of statistical heterogeneity. Rather, treatment effects need to be considered in relation to clinically relevant absolute effects, meaningful decision thresholds, and plausible effect modifiers. Importantly, inconsistency, indirectness, and imprecision represent interconnected dimensions that lower the certainty of the evidence. Geographic, healthcare system, or population differences become relevant when they plausibly alter or create tension among the key components of the *Population*, *Interventions, Comparators*, *and Outcomes* (PICO) framework. Together, these contributions move beyond the search for a single ‘universal truth’, encouraging a contextual interpretation of evidence while providing a conceptual framework for future clinical research.

The two papers related to the APIxaban Cancer-Associated Thrombosis (API-CAT) trial provide a practical illustration of the concepts detailed above. Mahé *et al.*^3^ explored how investigators approached extended anticoagulation in patients with cancer-associated thrombosis after the discontinuation of blinded study treatment and before trial unblinding and results. Clinicians’ decisions regarding continuation, discontinuation, and dose of direct oral anticoagulation varied according to treatment timing, physician speciality (thrombosis- vs. oncology-related specialists), and geography, despite similar clinical context. The accompanying viewpoint from Di Nisio *et al.*,^4^ underlines that API-CAT has provided clear evidence that reducing the dose of apixaban in patients with active cancer who had already completed at least 6 months of full-dose anticoagulation was non-inferior to a full dose for preventing recurrent venous thromboembolism, while being superior in terms of safety over 12 months of follow-up. However, challenging questions remain: who should continue anticoagulation, for how long, and how often should these decisions be re-assessed? These manuscripts continue previous discussions related to the duration of anticoagulation in cancer-associated thrombosis and the profile of patients requiring extended prophylaxis.^5^ Contemporary practice increasingly relies on individualized assessment of competing risks to balance thrombotic protection and bleeding risk. API-CAT-derived evidence reduces uncertainty regarding the optimal intensity of apixaban treatment, but several clinically relevant questions remain.

Evidence-based and patient-tailored decisions need drug adherence and persistence, especially in a polypharmacy and multimorbid context, to ensure long-term therapeutic benefit. Sandslätt *et al.*^6^ focus specifically on statin adherence among 30 497 patients with hypertension included in a large national registry. Using explainable machine-learning approaches, they identified substantial variation in persistence, measured as 2-year proportion of days covered of above 80%, which was achieved in only 60% of the patients. Persistence was associated with concomitant antithrombotic therapy, use of angiotensin-converting enzyme (ACE) inhibitors, increasing age, healthcare centre, socioeconomic status, and statin type. These findings highlight low statin persistence in high-risk individuals, especially in those in primary prevention, as an actionable target for improving cardiovascular prevention. A complementary Swedish nationwide study involving more than 150 000 post-MI patients showed that, although statin discontinuation was common, treatment re-initiation was also frequent, resulting in ∼91%–92% of patients remaining on treatment at 1 year and 74%–79% at 12 years after the index event.^7^ Together, these studies reinforce the notion that prescribing the right drug to the right patient at the right time is insufficient without helping to maintain drug use over time, with the healthcare system becoming an active player in this intervention.

Beyond drug use implementation, the pathophysiological features of the individual patient can affect drug pharmacokinetics (PK). Vlachakis *et al.*^8^ review the available evidence in patients with Fontan circulation and describe how Fontan-associated progressive hepatic and renal dysfunctions, protein-losing enteropathy, and altered haemodynamics profoundly affect all PK steps, namely absorption, distribution, metabolism, and elimination of the major drug classes used in these patients. Given the severity and the rarity of this condition, physiologically based pharmacokinetic modelling may be particularly helpful to integrate complex and progressive multisystem dysfunction with drug-specific PK,^9^ providing guidance for dose selection and reducing the risk of therapeutic failure or toxicity.

Shiba *et al.*^10^ addressed the toxicity of hydroxychloroquine, commonly prescribed for malaria and autoimmune diseases and generally considered safe, through an integrated translational study. In a retrospective cohort of 261 hydroxychloroquine-treated patients, though clinically relevant cardiac adverse events were uncommon, 14 individuals discontinued treatment due to cardiac adverse effects, including conduction abnormalities and biopsy-confirmed cardiomyopathy. *In vitro* experiments using human-induced pluripotent stem cell-derived cardiomyocytes exposed to the drug reproduced electrical abnormalities, contractile dysfunction, lysosomal changes, and impaired autophagic flux, providing mechanistic support to the clinical observations. These findings extend contemporary understanding of cardiotoxicity associated with non-oncologic medications and complement recent work emphasizing the importance of patient-specific susceptibility, comorbidities, and concomitant therapies in determining cardiovascular safety of non-oncologic drugs.^11^

To conclude, the leitmotif of this issue of the *EHJ-CVP* is that context, broadly defined, influences every stage of cardiovascular pharmacotherapy development and implementation. It shapes how evidence is generated and interpreted, how clinicians make decisions, whether patients persist with prescribed therapies, and ultimately how drugs are handled and tolerated in complex pathophysiological conditions that affect their PK and pharmacodynamics (PD). Precision pharmacotherapy, therefore, depends on contextualizing evidence, clinical decisions, patient behaviour, disease-related determinants of PK and PD, and healthcare systems. The challenge is no longer simply to identify universal truths but to understand the populations, interventions, comparators, outcomes, and clinical settings in which those truths remain valid.

## Data Availability

No data have been generated for this manuscript.
